# Real-Time Assessment of the Size Changes of Individual Sub-Visible Protein Particles under Buffer Variations: A Microfluidic Study

**DOI:** 10.3390/ph16071002

**Published:** 2023-07-14

**Authors:** Drago Kuzman, Urška Klančnik, Eva Grum, Jure Derganc

**Affiliations:** 1 Novartis d.o.o., Kolodvorska 27, 1234 Mengeš, Slovenia; drago.kuzman@novartis.com (D.K.); eva.gum@novartis.com (E.G.); 2 Institute of Biophysics, Faculty of Medicine, University of Ljubljana, Vrazov trg 2, 1000 Ljubljana, Slovenia

**Keywords:** aggregation, monoclonal antibody, biologics, microfluidics, optical tweezers, intermolecular forces

## Abstract

Protein particles in biological drugs can significantly impact drug efficacy and carry the risk of adverse effects. Despite advancements, the understanding and control of particle formation in biopharmaceutical manufacturing remain incomplete. Therefore, further investigation into protein particles is warranted, especially considering that novel formats of biological drugs may be more susceptible to aggregation and particle formation than conventional monoclonal antibodies. In this study, we introduce a microfluidic approach for the real-time analysis of individual sub-visible protein particles during buffer exchange. We find that the modulation of intermolecular forces, achieved by changing the buffer pH or urea concentration, leads to the reversible swelling and shrinkage of particles by up to 50%, which is a consequence of altered intermolecular distances. Additionally, we identify a discrepancy in the biophysical behavior of protein particles compared to monomeric protein. This finding highlights the limited predictive power of commonly applied biophysical characterization methods for particle formation in early formulation development. Moreover, the observed particle swelling may be associated with manufacturing deviations, such as filter clogging. These results highlight the importance of studying individual particles to gain a comprehensive insight into particle behavior and the impact of formulation variations in the biopharmaceutical industry.

## 1. Introduction

Since the first therapeutic monoclonal antibody (mAb) was approved by the FDA in 1986, mAb-based biologics have revolutionized the treatment of previously incurable diseases, including certain cancers and autoimmune diseases [1]. With more than 100 FDA-approved mAb products as of 2021 and ongoing research into new targets such as Alzheimer’s disease [2], the field continues to expand. However, the production of biopharmaceuticals presents several challenges, including issues related to protein aggregation into large protein particles that can adversely affect product quality, efficacy, and patient safety. A recent analysis of particle-related issues at Novartis highlighted the prevalence of large, visible protein particles [3], particularly in large-scale manufacturing. In addition, novel biologics formats (non-mAbs) are more prone to particle formation, highlighting the importance of a better understanding and control of biologics aggregation [4].

The formation of aggregates and particles through various degradation pathways leads to a diverse range of characteristics. These entities can arise in bulk solution or at interfaces and result from reversible protein association or conformational changes leading to irreversible oligomerization, nucleation-controlled aggregation, or surface-induced aggregation [5,6]. The aggregation of monoclonal antibodies (mAbs) can occur due to various chemical and physical factors during manufacturing, storage, or shipping. Several mechanisms contribute to variations in the size, number, and morphology of aggregates. These processes are governed by intermolecular forces that are also responsible for the tertiary structure of proteins, such as hydrogen bonds, Van der Waals attraction, hydrophobic and electrostatic interactions, or covalent disulfide bonds. Aggregates often grow considerably, first forming oligomers on the nanometer scale, then sub-visible particles with sizes up to several hundred micrometers, and eventually visible millimeter-sized precipitates. The analysis and characterization of these aggregates and particles is a major challenge due to their broad size range. Although many analytical techniques for particle characterization are available, the field is continuously evolving, and a comprehensive investigation of particle size and quantity in biopharmaceutical products often requires the integration of multiple complementary analytical methods.

Large protein particles are too big to be effectively studied by the traditional biochemical methods, such as size exclusion chromatography and dynamic light scattering, used to study small protein oligomers [7]. To study the aggregation of large particles, new methods have been developed that borrow from the physical and life sciences. For example, a method for assessing the aggregation of mAbs on the water–air surface based on Brewster angle microscopy was recently presented [8]. A large class of methods particularly suitable for large particles is based on microfluidics [9,10,11]. One of the most prominent examples of microfluidic methods is microflow imaging (also called flow imaging microscopy), which has become a standard tool in the development and production of biologics [7] (USP <1788.3>).

Microfluidics has emerged as a powerful tool for the characterization and formulation development of biological drugs, providing precise and efficient analysis in a miniaturized format. By manipulating small sample volumes, microfluidic platforms enable the study of critical parameters such as the protein–protein interactions [12], viscosity, and aggregation of biological drugs [13]. They facilitate rapid and parallel analysis and enable the comprehensive characterization of drug formulations and the evaluation of critical quality attributes. In addition, microfluidics can be integrated with sensitive detection methods that enable the real-time monitoring and analysis of drug samples, such as liquid cell electron-microscopy imaging [14].

In this work, we apply a microfluidic approach to study how individual sub-visible protein particles respond to changes in their surrounding buffer. Specifically, we monitored single mAb particles in a microfluidic diffusion chamber in real time during reversible changes in buffer pH and urea concentration, which affect intermolecular forces. We found that the particles swelled considerably when the pH was changed from the neutral value or when the urea concentration was increased. After the conditions were restored, the particles returned to their initial size and shape, indicating that the swelling was a consequence of increased intramolecular distances. Remarkably, the observed behavior of particles differed from the response of monomers to pH changes, analyzed by dynamic light scattering.

## 2. Results

### 2.1. Particle Manipulation

**Figure 1:**
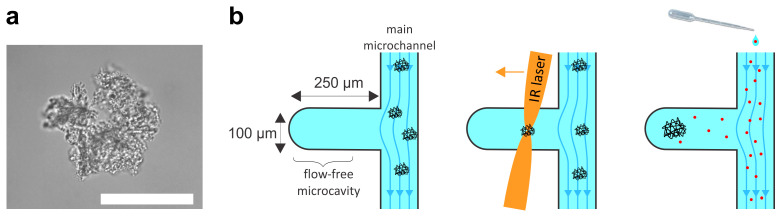
Experimental set-up. (**a**) A typical sub-visible protein particle prepared by stirring a solution of mAbs. The particles can be moved and rotated with optical tweezers as quasi-rigid bodies (Video S1). The scale bar indicates 50 μm. (**b**) Schematic representation of the experiments in the diffusion chamber. The chamber is a microcavity extending from the main channel of the microfluidic system. The length of the cavity is 250 μm, its width is 100 μm, and its depth is 40 μm. Protein particles are transferred into the chamber by optical tweezers and the buffer surrounding the particles in the chamber is then exchanged by diffusion from the main channel. The buffer in the main channel can be exchanged by a standard pipette. As the chamber is effectively flow-free, the particles remain undisturbed and can be monitored in real time during repeated buffer exchange.

Protein particles were prepared by stirring a solution of IgG1 mAbs with a magnetic stirrer and analyzed under a microscope with a WI 60 × 1.0 NA objective. The preparation yielded particles with a wide range of sizes and shapes. At the lower boundary, the size of particles detected was limited by the optical resolution of the microscope, i.e., at approximately 1 μm. Particles with diameters ranging from approximately 20 μm to 50 μm were selected for the analysis (Figure 1a). As expected, smaller particles exhibited Brownian motion, while larger particles appeared immobile. Using optical tweezers, the large particles could be slowly rotated and moved as quasi-rigid bodies (Video S1). The particles could thus be moved from the main channel of the microfluidic system into the diffusion chamber for subsequent buffer exchange (Figure 1b).

### 2.2. Particle Response to Changes in pH and Ionic Strength

**Figure 2:**
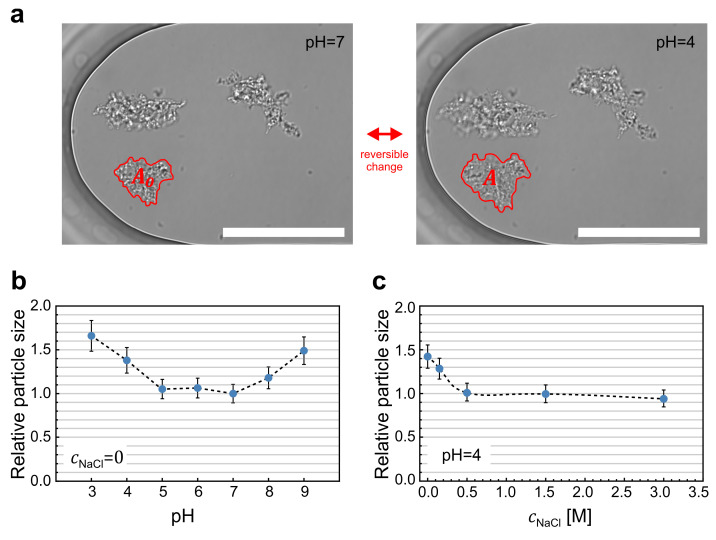
Response of protein particles to altered electrostatic interactions. (**a**) Three representative protein particles in the microfluidic diffusion chamber at the beginning of the experiment at pH 7 (left) and after the pH was changed to 4 (right). Particle size was quantified as the surface area of the particle outline (*A*) relative to the initial state (*A*_0_). Scale bars correspond to 50 μm. (**b**) Dependence of the relative size of protein particles on the value of pH in the absence of NaCl. All the changes were reversible, except at pH 3, at which the particles irreversibly decayed. (**c**) Dependence of the relative size of protein particles on the concentration of NaCl at pH 4.

First, we tested if the mAb particles are influenced by intermolecular electrostatic forces. The particles were transferred into the diffusion chamber in the initial buffer with pH 7 and low ionic strength. Then, the buffer in the chamber was successively replaced by buffers with pH values ranging from 3 to 9. Importantly, these buffers did not contain mAbs. The particles responded to the pH changes by changing their size in real time (Figure 2a and Video S2). The particle sizes were quantified by measuring the surface area of the particle outline on the microscope image using ImageJ software (Figure 2a). The results are shown in Figure 2b. The particle size increased by more than 50% when the pH of the solution deviated from the neutral value. Notably, the particle shape remained unchanged during its size changes (Figure 2a), indicating that particle swelling was a result of increased intermolecular repulsion forces and not due to additional monomers aggregating onto the particle. For small deviations in pH, all changes in particle sizes were reversible (Figure 2a and Video S2). On the other hand, changing the pH to 2 caused the particles to break down (Video S3). Changing the pH back from 2 to 7 reinduced the protein aggregation, but the newly formed particles were smaller and connected into a mesh-like structure (Video S4).

To further investigate the presence of electrostatic forces in the protein particles, we increased the concentration of NaCl to screen the surface charge. The experiment was performed at pH 4, at which the particles reached a stable maximum size (Figure 2b). After increasing the ionic strength in the buffer at pH 4 to approximately 0.5 M NaCl, the protein particles shrank back to their initial size (Figure 2c), i.e., from a relative size of 1.4 to 1.

**Figure 3:**
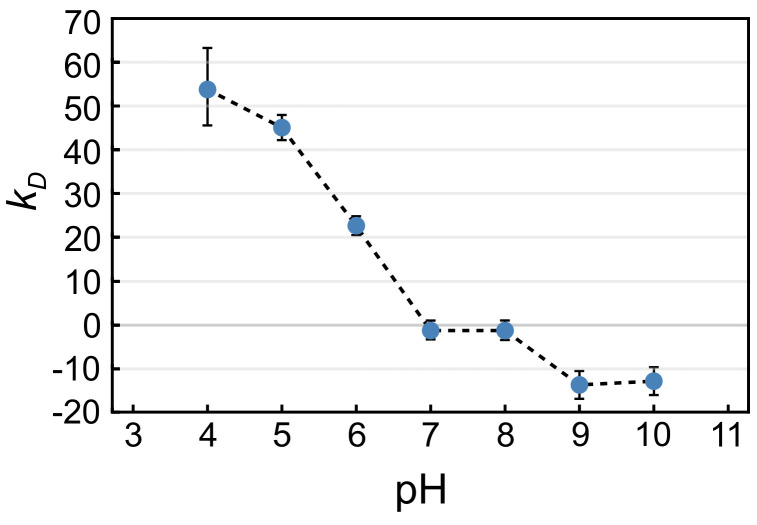
Effect of pH on the protein-protein interaction coefficient (*k_D_*) as measured by dynamic light scattering; *k_D_* is a measure of protein–protein interaction: *k_D_* > 0 repulsive interaction, *k_D_* < 0 attractive interaction.

To compare the response of protein particles with the behavior of monomers, the interaction between monomers was assessed by calculating the protein–protein interaction coefficient (*k_D_*), which was determined as the slope of the protein diffusion change with respect to protein concentration [15,16,17]. The results revealed repulsive interactions at low pH, transitioning into weak attractive interactions near the isoelectric point (Figure 3). Additionally, it was observed that increasing the ionic strength of the solution resulted in a decrease in repulsive interactions. Note that the behavior of monomers reflects the value of the isoelectric point at a pH of around 8, while the repulsive interactions in particles in Figure 2b were minimized in the pH range of 5 to 7 (Figure 2b).

### 2.3. Particle Response to Urea

**Figure 4:**
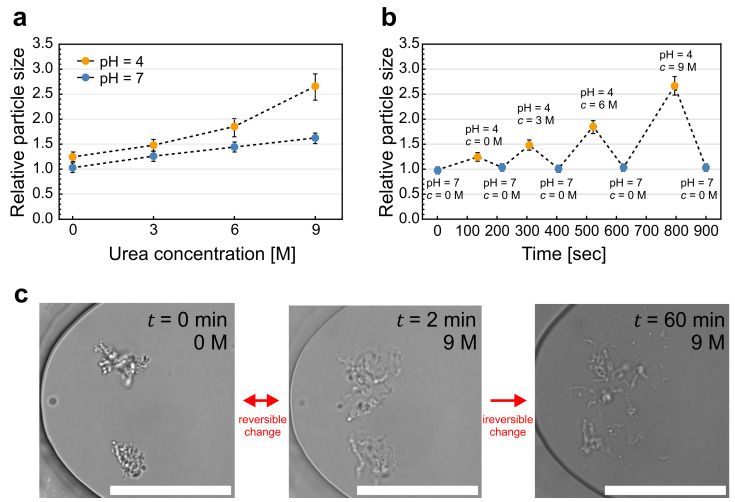
Response of protein particles to urea. (**a**) Dependence of the relative particle size on urea concentration at pH 7 (blue) and pH 4 (yellow). (**b**) Reversible changes in particle sizes as the buffer was cyclically changed from 0 M urea at pH 7 to 0 M urea at pH 4, 3 M urea at pH 4, 6 M urea at pH 4, and, finally, to 9 M urea at pH 4. (**c**) Rapid changes between 0 M and 9 M urea are reversible, but if the particles are left in 9 M urea for a longer time, they disintegrate irreversibly. The scale bars represent 50 μm.

To investigate the impact of cold denaturation on protein particle size, we utilized the chemical denaturant urea due to its higher solubility compared to the commonly used guanidine hydrochloride. Both urea and guanidine hydrochloride can induce the reversible denaturation of proteins by disrupting the non-covalent interactions responsible for maintaining the protein’s folded structure. The subsequent removal of the denaturant allows for the potential refolding of the protein back into its native conformation. In our study, we exposed the particles to urea solutions with concentrations up to 9 M, which is the maximum attainable concentration in water [18]. Additionally, the experiments were carried out at two pH values. First, at pH 4, where the largest reversible increase in particle size was detected in the absence of urea with no noticeable particle disintegration (Figure 2b). Second, at pH 7, where no particle swelling due to electrostatic repulsion was observed and the particles maintained their minimum size in the absence of urea. The results showed that in both cases, the particles swelled with increasing urea concentration, as shown in Figure 4a. At pH 7, the particle size increased by more than 50%, and at pH 4, the particles swelled by more than 150% of their initial size. Remarkably, the latter effect was greater than the sum of the individual effects of electrostatic repulsion at pH 4 and urea alone.

To test the reversibility of particle swelling due to urea, we changed the buffer cyclically from pH 7 without urea to pH 4 with increasing concentrations of urea (Figure 4b). The entire experiment lasted approximately 15 min. Again, the swelling of particles was reversible (Figure 4b,c) unless they were left in the highest urea concentration for an extended period. In this case, they disintegrated in a similar manner as at the extreme pH values (Figure 4c). During all the reversible changes, the particles retained their original shape, indicating that the swelling was a consequence of increased intermolecular forces rather than the aggregation of additional monomers.

## 3. Discussion

Protein particles are composed of thousands of aggregated proteins, making the problem of their aggregation even more complex than that of protein folding. It is therefore not surprising that understanding and controlling the aggregation of proteins into protein particles remains a significant challenge in the production of biologics, a challenge similar to the unresolved issues related to amyloid aggregation in neurodegenerative disorders. Due to the substantial size of sub-visible protein particles, traditional methods of molecular biochemistry are insufficient for their assessment, necessitating the use of novel techniques better suited for the mesoscopic scale.

In this paper, we have presented a microfluidic approach to the study of individual sub-visible mAb particles during reversible changes in their surrounding buffer. To the best of our knowledge, this is the first study to show that the size of protein particles dynamically responds to buffer changes that influence intermolecular forces and, hence, the intermolecular distances within the particle. Specifically, the particles readily swell in buffers with altered pH or with increasing urea concentration even when the buffer does not contain free mAb monomers. For moderate changes in pH and urea concentration, the particle shape remains unaltered, and the changes are reversible.

The aggregation of mAb is a complex process that depends on all intermolecular forces that also stabilize the tertial structure of proteins, i.e., electrostatic and hydrophobic interactions, Van der Waals attraction, and hydrogen bonding [4,19]. Hence, the aggregation propensity of different types of mAbs can be markedly different and depends subtly on their structure [20]. When mAbs are near the native state, they can reversibly self-aggregate into oligomers. If they are denatured due to chemical or physical stress, new “hot spots” for intermolecular forces are exposed and mAbs begin to irreversibly aggregate into larger particles that eventually precipitate [5].

The presented study focused on the behavior of already formed particles and is, therefore, complementary to the standard biochemical studies of mAb aggregation [20,21,22,23]. The latter showed that aggregation can be triggered by a pH shift, which denatures the mAbs. The aggregation can be further enhanced by the addition of salt, which screens the surface charge, reduces the electrostatic repulsion, and facilitates contact between adjacent hot spots. Our study has shown that electrostatic interactions remain important even after the particles are formed. A shift in pH towards acidic or basic conditions resulted in an increase in particle size, with the smallest size observed in the pH range between 5 and 7 (Figure 2b). This swelling phenomenon can be attributed to the presence of charges of the same sign on the protein surface (positive at a lower pH, and negative at a higher pH), which increased net electrostatic repulsion and consequently increased the intermolecular distances within the particle. When salt was added to the solution, it screened the surface charge, and the particles shrank back to the initial size (Figure 2c). For small pH shifts, the particle behavior was reversible, which agrees with the nature of electrostatic interactions.

It is noteworthy that the observed phenomena could not be accurately predicted based on the properties of the monomers such as the protein–protein interaction parameter (*k_D_*) and the isoelectric point of the monomeric protein, which are usually determined during the early formulation development of biologics. The isoelectric point of the studied mAbs was around 8 (Figure 3), and one might expect symmetrical swelling of protein particles around this point. This, however, was not confirmed in our study. Similarly, the increase in particle size at pH 8 and higher could not be predicted solely by *k_D_* measurements alone, suggesting the involvement of attractive protein–protein interactions in a basic environment. These discrepancies between the behavior of the monomeric protein and the large protein particles indicate distinct biophysical properties, likely due to differences in the structure and distribution of the exposed amino acid residues on their respective surfaces.

The effect of urea on protein stability is not yet fully understood as it is extremely complex and can affect several intermolecular forces [24,25]. Our experiments showed that protein particles swelled considerably upon the addition of urea, indicating the reduction in intermolecular attractive forces by urea. The observed size increase with urea at pH 4 was greater than the sum of these effects separately (Figure 4), further illustrating the delicate balance between attractive and repulsive intermolecular forces within protein particles. Unfortunately, the optical resolution did not allow us to distinguish whether this swelling was a consequence of the swelling of individual proteins or a result of the reduction in inter-protein aggregation forces. In any case, the effect of a moderate urea concentration on protein particles was reversible. The irreversible disintegration of the particles was observed only in the presence of large pH shifts or in 9 M urea for extended periods. Clearly, these extreme conditions did not break down the particles to native monomers, as evidenced by the prompt reappearance of smaller particles once initial conditions were restored.

The presented study focused on the effects of pH and urea on sub-visible protein particles, but the approach could also be readily employed to assess the effects of the reagents used in the biopharmaceutical industry. For example, our preliminary experiments with sucrose, maltose, trehalose, sorbitol, and PEG at concentrations up to 200 mM indicate that these substances do not induce significant changes in protein particle size, presumably because their impact on intermolecular forces is limited. Moreover, the presented technique could be extended by including additional microfluidic components to simulate various process stresses, such as temperature excursions, interfacial stress, and freeze–thaw cycles. In this way, it is possible to comprehensively investigate the effects of these conditions on the behavior of protein particles and test whether particles induced by different mechanisms have different biophysical properties. In addition, the throughput of the method could be significantly improved by using advanced machine learning algorithms for particle evaluation [26] instead of relying on the manual measurement used in the present study. The enhanced capabilities will allow a deeper understanding of the impact of different manufacturing, storage, or shipping conditions on particle dynamics. With future technological advances, the approach could also be combined with advanced methods for liquid electron microscopy [14] to provide insight into particles too small to be resolved with optical microscopy.

## 4. Conclusions

In summary, this paper addresses persistent challenges of particle formation within the biopharmaceutical industry by proposing a novel application of optical tweezers and the microfluidic chamber for the manipulation and analysis of single sub-visible protein particles. The approach enabled the real-time evaluation of the size changes of individual protein particles due to altered intermolecular forces and revealed a distinct biophysical behavior in larger protein particles compared to monomeric proteins, providing valuable insights into the limitations of conventional biophysical characterization techniques. Moreover, the study highlights the potential correlation between manufacturing deviations, such as filter clogging, and observed particle swelling phenomena. This underscores the need to study and optimize formulation processes to ensure the quality and safety of biological drugs. The diffusion chamber with integrated optical tweezers represents a unique and promising opportunity for advancing the field of single-particle analysis.

As a complement to the existing arsenal of biochemical and biophysical methods for assessing protein aggregation, the microfluidic approach presented will facilitate the development of strategies to mitigate particle-related problems and improve the efficiency and reliability of pharmaceutical manufacturing processes. Ultimately, such studies will help improve quality control and ensure optimal therapeutic outcomes for patients receiving biopharmaceutical treatments [23].

## 5. Materials and Methods

### 5.1. Reagents and Protein Particle Preparation

The stock solution containing monoclonal antibodies type IgG1 at a concentration of 19.5 mg/mL was provided by Lek Pharmaceuticals d.d. (member of Novartis), and the other reagents were purchased from Sigma (St. Louis, MI, USA): urea (51456), Pluronic F 127 (P2443), HEPES (H3375), BSA (A3294), and PBS (P4417). The mAb particles were prepared by stirring the mAb stock solution on a magnetic stirrer for 2 h @400 RPM at room temperature. The pH of the stock solutions was 7. The isoelectric point of mAb was approximately 8.

### 5.2. Microfluidic Diffusion Chamber

The response of protein particles was monitored in a microfluidic diffusion chamber, which is described in detail in Vrhovec et al. [27,28]. Briefly, the microfluidic system was constructed by standard soft-lithography methods. The diffusion chamber was designed as a simple cavity extending sideways from the main microfluidic channel (Figure 1b). The cavity was 250 μm long, 100 μm wide, and 40 μm deep. The design mask was printed using a high-quality laser printer and the master mold was etched into a 4-inch silicon wafer using UV lithography and a photoresist (SU-8 2025, micro resist technology GmbH, Nüremberg, Germany ) to a total depth of 40 μm.

Transparent polydimethylsiloxane polymer (Sylgard 184, Dow Corning, MI, USA) was poured over the master mold to a thickness of approximately 5 mm and cured at 120 °C for 25 min. The solidified polymer cast was peeled off the mold and cut. The cast was then pierced at the entrance to create a 7 mm-wide entrance reservoir open to the air and pierced at the exit to connect the tubing leading to the output reservoir. After 45 s of the activation of both surfaces by air plasma generated in a plasma cleaner (PDC-002-CE, Harrick Plasma, NY, USA), the polymer cast was sealed on a glass coverslip (24 mm × 60 mm) and immediately wetted with distilled water. After wetting, the exit hole of the system was connected to the output reservoir filled with distilled water and mounted on a digital caliper (Absolute Digimatic Height Gauge 570-304, Mitutoyo, Japan).

The microfluidic chip was mounted on an inverted microscope (Nikon Ti-U, Tokyo, Japan) equipped with a 60× WI 1.0 NA objective and optical tweezers (λ = 1064 nm, Tweez, Aresis d.o.o., Slovenia). The entrance reservoir of the system was open to the air so that the solution flowing through the system could be exchanged using a standard pipette. Before introducing the protein particles, the system was washed with a solution of 40 μM Pluronic F 127 + 0.1M HEPES + 1.5 M NaCl and then with a buffer containing 20 mM PBS + 0.5% BSA to prevent the excessive adsorption of the protein on the microchannels. Note that BSA was used only for coating the microchannels prior to the experiments with protein particles and was absent in all subsequent experiments with mAbs.

Flow through the main channel was controlled by adjusting the height of the output reservoir. A 10 cm height difference between the entrance and output reservoirs resulted in average flow velocities of about 0.5 mm/s in the main channel, i.e., flow rates of approximately 2 nL/s. The maximum height difference attainable with the height gauge was approximately 40 cm.

It has been shown that the fluid flow from the main channel does not enter the diffusion chamber [27,29] and that solute exchange between the chamber and the main channel occurs solely by diffusion. Specifically, the flow rate in the chamber decays exponentially with a decay length of approximately 30 μm [29]. At 100 μm into the chamber, the flow rate is only approximately 1/100th of the maximal flow rate in the main channel. The experiments with protein particles were conducted at the far end of the chamber, approximately 200 μm from the main channel, where the flow rate is 10,000 smaller than in the main channel. Thus, the back side of the chamber can be considered effectively flow-free. The exchange time for solute diffusion between the back side of the chamber and the main channel is typically in the order of minutes, depending on the solute’s molecular weight, e.g., for small molecules with $M_{W}\sim$300 g/mol and $D\sim 5.7\;10^{-6}\;{\mathrm{cm}}^{2}/s$, the exchange time is less than one minute, and for large proteins such as hemoglobin with $M_{W}\sim$60,000 g/mol and $D\sim 9.4\;10^{-6}\;{\mathrm{cm}}^{2}/s$, the exchange time is less than 5 min [27]. Hence, one can expect that the buffers with different pH values and urea concentrations exchange in less than one minute, while mAb monomers need more than 5 min to diffuse into or out of the chamber.

### 5.3. Measurements of Relative Particle Sizes

After the solution containing protein particles was introduced into the main channel, at least three protein particles with diameters of >20 μm were selected and transferred to the diffusion chamber using optical tweezers (Figure 1b). The solution in the main channel was then changed and the response of the protein particles in the chamber was monitored in real time as the new solution diffused into the chamber. The solution in the main channel could be changed using a standard pipette without disturbing the particles in the diffusion chamber so that the response of individual particles could be followed across many solution exchanges. The experiments were conducted at room temperature (22 °C). The protein particles in the diffusion chamber were recorded with a Pixelink PL-B741U monochrome digital camera using software provided by the manufacturer of the optical tweezers.

The relative particle size (a) was quantified as the surface area of the particle outline on the microscope image (A) divided by its initial surface area ($A_{0}$), as presented in Figure 2a:

(1) $$a=A/A_{0}$$

The surface area of the particle outline was determined using ImageJ software v 1.53c [30]. First, the contour around the particle was drawn manually using the freehand selection tool, and then the built-in measurement function was applied to determine the surface area of the selection (A). Note that the relative particle size defined in this way is related to the surface area of the particle outline. Assuming that the change in particle size is the same in all directions, the relative particle diameter is the square root of this number and the relative particle volume is equal to $a^{3/2}$. To verify the repeatability of the method, a single particle was measured repeatedly three times under the same buffer conditions. The measurement error for the method was found to be in the order of 5%.

With each solution in the diffusion chamber, at least three different particles were measured, and the result was given as the mean value ± SD.

### 5.4. Protein–Protein Interaction Coefficient k_D_

The protein–protein interaction coefficient *k_D_* of mAbs monomers in solution was determined by the method described in [15,16,17]. Briefly, DLS analysis was conducted using Malvern Instruments’ APS S at 25° ± 0.1 °C. Each sample was subjected to 15 scans with a duration of 5 s per scan. The acquired correlograms (correlation function versus time) were analyzed using Malvern software to calculate the diffusion coefficient. The protein–protein interaction coefficient *k_D_* was determined for each pH separately by fitting the following equation:

(2) $$D=D_{0}\left(1+k_{D}c_{p}\right),$$

where *D*_0_ is the diffusion coefficient at infinite dilution and *c_p_* is the protein concentration. Diffusion coefficient *D* was measured at several protein concentrations *c_p_* in a range from 1 to 6 mg/mL. Based on DLS experiments at a concentration of 1 mg/mL, the apparent hydrodynamic radius of mAbs at pH 3 was estimated to be about 5.5 nm, while the radius at pH 9 was about 9 nm. All samples were analyzed in triplicate and the result is given as the mean value ± SD.

## Data Availability

Data is contained within the article.

## Supplementary Materials

The following supporting information can be downloaded at: https://www.mdpi.com/article/10.3390/ph16071002/s1. Video S1: Manipulation of an mAb particle with optical tweezers (the particle is the same as presented in Figure 1a). The small black cross indicates the position of the optical trap when the trap is turned on. Video S2: Three mAb particles as they swell and shrink during pH shift from 7 to 4 and back to 7. Video S3: Three mAb particles as they disintegrate when the pH was shifted from 7 to 2. Video S4: The continuation of the experiment presented in Video S3 as the pH was shifted from 2 back to 7.
